# Behavioral pathway to a broken heart: The link between adverse childhood experiences, depression, physical exercise and cardiovascular health

**DOI:** 10.3389/fpsyt.2022.1002143

**Published:** 2022-10-11

**Authors:** Sebastian Bertele, Ivo Heitland, Daniela Fraccarollo, Britta Stapel, Johann Bauersachs, Mechthild Westhoff-Bleck, Kai G. Kahl

**Affiliations:** ^1^Department of Psychiatry, Social Psychiatry and Psychotherapy, Hanover Medical School, Hanover, Germany; ^2^Department of Cardiology and Angiology, Hanover Medical School, Hanover, Germany

**Keywords:** childhood trauma questionnaire, childhood adversity, childhood maltreatment, cardiovascular disease, depression, physical activity, exercise, epicardial adipose tissue

## Abstract

**Background and aim:**

Adverse childhood experiences (ACEs) are a major risk factor for unfavorable behavioral, mental and health outcomes later in life. However, the precise pathway *via* which ACEs convey these risks, in particular regarding health outcomes such as cardiovascular disease, remains unknown. Here, we combined psychiatric and cardiac methods to investigate the pathway *via* which childhood adversities may lead to adult adverse cardiovascular health, with a focus on epicardial adipose tissue (EAT) as a risk marker.

**Methods:**

210 adult congenital heart disease outpatients (mean age 35.5 y, 43% female) completed a thorough cardiac and psychiatric evaluation. Psychiatric measurements included an expert interview, the childhood trauma questionnaire (CTQ), Beck's depression inventory II (BDI-II), quality of life and the global scale of functioning, amongst others. All patients completed a full cardiac workup including EAT assessment using echocardiography. We then computed bootstrapping mediation models using ACEs as a predictor, depression and physical activity as mediators and EAT as dependent variable in PROCESS.

**Results:**

CTQ scores had a significant indirect effect on EAT *via* a serial mediation of BDI and physical activity [a^*^b2^*^d = 0.0260, 95% BCa CI [0.0047, 0.0619]].

**Conclusion:**

Using mediation analyses, we show that adverse childhood events are linked to increased depressive symptoms, which are linked to decreased physical activity, which in turn are linked to a higher amount of epicardial adipose tissue. While other pathways most certainly exist and replication is needed, this suggests a meaningful pathway *via* which ACEs lead to adverse cardiovascular health, with several potential targets for health interventions across time.

## Introduction

Adverse childhood experiences (ACE) are common (1, 2) and pose a high risk for adverse mental health effects (3–5). Emotional or physical abuse and neglect alter what is considered normal psychological development, resulting in limitations of varying degree in mentalization (6), regulation of emotion (7–9), and social interaction (4, 8, 10). All these factors increase a person's likelihood to develop depression or depressive symptoms later in life (2, 10).

ACE such as emotional and physical abuse have long been linked to adverse mental health in adulthood (e.g., major depressive disorder (MDD), post-traumatic stress disorder, anxiety disorder, psychotic disorders) (3, 11, 12) and, more recently, to an earlier decline in cardiovascular health (11, 13) with higher rates of adverse cardiovascular events (e.g., myocardial infarction, coronary artery disease and stroke) (14). Current evidence suggests MDD as an independent risk factor for cardiovascular disease (CVD) (15, 16).

ACE, as described above, are a risk factor for MDD (10). Not only does MDD affect the prevalence of adverse cardiovascular events, it has also been established that depressive symptom have a negative impact on the long-term outcome of those who suffer from CVD (16). A study by Lespérance et al. showed that 5-year mortality after myocardial infarction was higher for those who showed signs of depression (12, 17, 18). Similarly, it has been shown that adult congenital heart disease (ACHD) patients who suffered from MDD showed increased epicardial tissue, a risk marker for the development of CVD (19–21), when compared to those without MDD (22). It therefore stands to reason that there is a link between ACE and cardiac health, potentially through the route of MDD or its psychopathological components.

Moreover, ACE lead to epigenetic (23) changes. They alter the hypothalamus-pituitary-adrenal axis (HPAA) (13), leading to a more pronounced stress reaction in those who have suffered from ACE. Further, increased activation of the sympathetic nervous system and higher activity of the amygdala and limbic system have been described (13). While dysregulation of endocrine and other systems is one possible route to explain the deleterious pathway linking ACE with CVD, other mechanisms may also play a role. Psychological changes (24) concurrent with depressive symptoms like lack of energy or loss of interest may favor an unhealthy lifestyle, which in turn may increase the likelihood of CVD. Behavioral alterations observed with ACE comprises higher rates of tobacco dependence, unhealthy nutritional choices and lack of physical exercise.

One of the generally accepted beneficial behavioral factors to cardiovascular health is physical activity. Regular moderate exercise benefits endothelial function and blood flow (25–27) and reduces general body inflammation (28). Additionally, numerous studies have shown beneficial effects of physical exercise on existing cardiac conditions (26, 27, 29–31). Hence, the absence of physical exercise may be considered a behavioral risk factor for adverse cardiovascular outcomes.

Most current research focuses on the relationship of two variables only–be it the connection between ACE and MDD (24, 32, 33), or the link between physical exercise and CVD (28, 29, 31). Some studies demonstrate links between ACE and CVD (11, 34, 35), and MDD has long been established as an independent risk factor for CVD (15, 16, 36, 37). Research investigating the moderating factors of this connection has thus far been scarce and focused mostly on biochemical and epigenetic changes (34, 38–41). Questions as to what factors mediate the correlation between ACE and CVD, or MDD and CVD remain unclear. The understanding of this pathway may lead to the use of more targeted and effective interventions in patients suffering from both MDD and CVD, and imply useful psychological and behavioral screening measures in the evaluation of cardiovascular risk.

This study examines such a possible pathway between ACE and unfavorable cardiovascular outcomes, and hypothesized that there is a direct behavioral pathway where MDD and subsequent lack of physical exercise moderate higher risk for adverse cardiovascular outcomes, particularly epicardial adipose tissue which is an established risk marker for cardiovascular events.

## Materials and methods

### Participants and study design

The data shown here are part of the PSYConHEART study, an ongoing research project investigating the morbidity and mortality factors in cardiovascular disease, and adults with congenital heart disease (ACHD) in particular. Parts of the data and the study protocol have been published earlier (22). All study procedures were approved by the local ethical committee of Hanover medical school. Written informed consent in accordance with the Declaration of Helsinki was provided by all subjects. All patients were recruited from the ACHD outpatient clinic of the Dep. of Cardiology and Angiology at the Hannover Medical School in Hannover, Germany. Inclusion criteria were (1) structural congenital heart disease, (2) ability to read and complete the informed consent form and questionnaires in German, and (3) age of 18 or older. Exclusion criteria were instable cardiac condition and pregnancy. The sample comprised two-hundred fifteen ACHD patients (120 males, 90 females) of whom 21 had to be excluded due to incomplete data. Details of the underlying heart diseases and treatments are given in Table 1 as well as a previous publication (22).

**Table 1:** Sociodemographic and cardiological data of the sample population.

	**Total *N* = 194**
Female gender	85 (43.8%)
Age	35.1 (±11.1)
BMI	25.4 (±4.98)
Drinks per week	1.96 (±3.44)
Smoker	52 (26.8%)
In Partnership	110 (56.7%)
**Schoolyears**	
Up to 9 years	31 (16.0%)
Up to 11 years	93 (47.9%)
Up to 13 years	70 (36.1%)
Currently Working	161 (83.0%)
**NYHA Class**	
I	149 (76.8%)
II	34 (17.5%)
III	11 (5.7%)
IV	0 (0%)
LVEF (in %)	56.8 (±8.62)
EAT	0.433 (±0.210)
**Congenital heart defect**	
Simple shunts	15 (7.7%)
Atrioventricular septal defect	8 (4.1%)
Mitral valve disease	4 (2.1%)
Anomalous pulmonary venous connection	1 (0.5%)
Bicuspid aortic valve	26 (13.4%)
Subaortic stenosis	6 (3.1%)
Coarctation	23 (11.9%)
Congenital pulmonary stenosis	8 (4.1%)
Double chambered right ventricle	2 (1%)
Tetralogy of fallot	29 (14.9%)
Ebstein anomaly	6 (3.1%)
Marfan syndrome	16 (8.2%)
D-Transposition: Atrial switch	24 (12.4%)
D-Transposition: Arterial switch	1 (0.5%)
Congenital corrected transposition	3 (1.5%)
Fontan type circulation	16 (8.2%)
Eisenmenger syndrome	6 (3.1%)

### Assessment of psychiatric disorders and behavioral factors

The psychiatric diagnosis was based on the Structured Clinical Interview for Diagnostic and Statistical Manual of Mental disorders, 4th edition (SCID) (42). Raters were experienced psychologists or psychiatrists and blinded for all cardiac data obtained from the patients. All participants underwent full SCID workup. Patients were included in analysis regardless of other diagnoses, like e.g., substance-abuse-disorders or psychotic disorders. Depressive symptoms were assessed using the Beck's depression inventory II (BDI-II) (43), depression severity was assessed using the Montgomery-Åsberg Depression Rating Scale (MADRS) (44). Furthermore, participants completed a demographic survey that included educational, marital, employment status, smoking habits (expressed as pack-years), and alcohol drinking behavior (expressed as drinks consumed per week). Physical activity and exercise were assessed using a 6-point Likert scale with descriptors described as “no physical activity or exercise training” (1); “occasional physical activity (such as walking) or exercise training” (2); “light physical activity or exercise training, but <1 × weekly (3); moderate physical activity or exercise: regular physical activity (cycling or walking) or exercise training 1 × weekly (4); often, more than 1 × exercise training weekly, or cycling plus regular walking; and “very often, exercise training more than 3 × weekly”(5) “daily, exercise training” (6) (45).

### Assessment of adverse childhood experiences

The extended German version Childhood Trauma Questionnaire (CTQ) (46, 47) was used to assess ACE. The 32-item self report questionnaire measures a total of seven subscales on a five-point Likert-Scale. The subscales are emotional abuse, emotional neglect, physical abuse, physical neglect, sexual abuse, Inconsistency, and, as part of the extended version of the questionnaire, “playing down” which measures the patients' tendency to downplay extreme experiences as normal.

For analysis in the pathway analysis only the total mean CTQ-Score was used.

### Assessment of cardiac disease

Each patient was thoroughly examined by a cardiologist as described before (22). Functional status was determined according to the New-York Heart Classification (NYHA class) (48). In short, echocardiography was performed in all patients to evaluate cardiac morphology and function. Cardiac defects were categorized as simple, of moderate or of great complexity using the Warns classification (49). Echocardiographic assessment of EAT was derived from two-dimensional standard parasternal long axis/short axis views at end-diastole. EAT thickness was measured on the right free ventricular wall perpendicular to the aortic annulus. The cardiologist performing echocardiographic assessments (M.W.-B.) was blind to all psychiatric data.

### Statistical analyses

**Figure 1:**
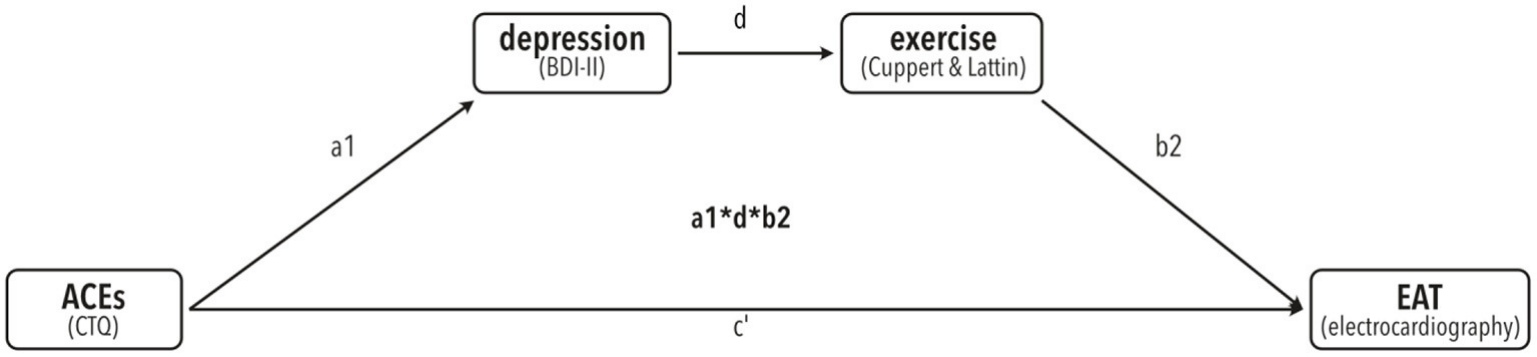
A-priori proposed mediation model of the link of ACE's (predictor) with EAT (dependent variable) *via* serial mediators depression (BDI-II) and exercise (Cuppett & Latin scale).

All statistical analyses were conducted with SPSS Statistics version 26.0 (IBM Corp., Amonk, NY, USA) (50) and R “Bird Hippie” V4.1.2 (51). An alpha of 0.05 was used for all statistical tests. Mediation analysis was performed by calculating bias-corrected accelerated (BCa) 95% confidence intervals (CIs) using bootstrapping with 10,000 resamples *via* the PROCESS procedure V3.4 for SPSS (52–54). To test our a-priori hypothesis that ACE's are linked to BDI, which is linked to physical activity, which is linked to EAT, we used a serial mediator model (number 6) with CTQ score as predictor, BDI and physical activity as mediators (in that order), and EAT as dependent variable. For an overview of the model tested here, see Figure 1.

In addition to this bootstrapping-based mediation analysis, following current guidelines (52), we performed regression analyses of all single paths of our mediation model to illustrate single-path links.

## Results

### Sample characteristics

For an overview of demographic and cardiovascular characteristics of the sample, see Table 1. The total sample included in statistical analysis included 194 patients with a mean age at the time of evaluation of 35 years (SD = 11.1 years), of which 85 (43.8%) were female. Less than a third of the patients were smoking at the time and a rough two thirds were in a romantic relationship or married. More than three quarters of the patients were currently working a job.

The sample's mean left ventricular ejection fraction was fairly good at 56.8% (SD = 8.62%) and symptoms of heart failure–especially dyspnoea–were low for most and moderate for some. None of the patients included in the analysis suffered from a NYHA grade IV heart failure.

All results from SCID-diagnostics are presented in Table 2. For characteristics of psychometric properties relevant to this study please refer to Table 3. The mean score from BDI-II was 7.06 (SD = 8.74) and, thus, quite low. However, 40 Patient's scored above the cut-off-level of 14 for a minor depressive Episode (respectively, 8.8% had minor depression, 8.8% moderate depression and 3.1% major depression). This was congruent with results of the semi-structured interview for DSM-IV (55) where 24.2% of the patients could be diagnosed with major depressive episodes and 38.7% patients fulfilled criteria of having suffered from depression within their lifetime. Only 15.6% reported a parent suffering from Depressions. For a more detailed report on the psychiatric characteristics of the sample population, please refer to earlier publications (56).

**Table 2:** Complete results from SCID-Diagnostics as *n* (% of *N*).

	**Total *N* = 194**
Current major depression	47 (24.2%)
Lifetime major depression	75 (38.7%)
Chronic depression	25 (12.9%)
Dysthymia	15 (7.7%)
Adjustment disorder	2 (1%)
Hypomania	3 (1.5%)
Delusional disorder	0 (0%)
Schizophrenia	0 (0%)
Substance abuse disorder	10 (5.2%)
Specific phobias	14 (7.2%)
Panic disorder with agoraphobia	5 (2.6%)
Agoraphobia	7 (3.6%)
Panic disorder without agoraphobia	11 (5.7%)
Social phobia	5 (2.6%)
OCD	3 (1.5%)
Bipolar I	1 (0.5%)
Bipolar II	2 (1%)
Generalized anxiety disorder	14 (7.2%)
PTSD	4 (2.1%)
Somatoform disorder	6 (3.1%)
Eating disorders	6 (3.1%)
ADHD	6 (3.1%)
Dyslexia	15 (7.7%)
Avoidant personality disorder	19 (9.8%)
Dependent personality disorder	11 (5.7%)
Anancastic personality disorder	27 (13.9%)
Negativisticpersonality disorder	0 (0%)
Depressive personality disorder	0 (0%)
Paranoid personality disorder	0 (0%)
Schizotypical personality disorder	1 (0.5%)
Schizoid personality disorder	0 (0%)
Histrionic personality disorder	0 (0%)
Narcicistic personality disorder	3 (1.5%)
Borderline personality disorder	18 (9.3%)
Antisocial personality disorder	1 (0.5%)

**Table 3:** Data relevant for the pathway-analysis.

	**Total *N* = 194**
Sportscore	
1	40 (20.6%)
2	29 (14.9%)
3	33 (17.0%)
4	35 (18.0%)
5	34 (17.5%)
6	23 (11.9%)
BDI-2	7.60 (±8.74)
0–8 no depression	122 (62.9%)
9–13 minimal depression	32 (16.5%)
14–19 minor depression	17 (8.8%)
20–28 moderate depression	17 (8.8%)
29–63 severe depression	6 (3.1%)
CTQ	40.5 (±13.2)
Emotional Abuse	7.42 (±3.53)
None to minimal	144 (74.2%)
Low to moderate	32 (16.5%)
Moderate to severe	7 (3.6%)
Severe to extreme	11 (5.7%)
Physical Abuse	5.89 (±2.50)
None to minimal	173 (89.2%)
Low to moderate	10 (5.2%)
Moderate to severe	4 (2.1%)
Severe to extreme	7 (3.6%)
Sexual Abuse	5.68 (±2.59)
None to minimal	174 (89.7%)
Low to moderate	4 (2.1%)
Moderate to severe	10 (5.2%)
Severe to extreme	6 (3.1%)
Emotional Neglect	8.93 (±3.91)
None to minimal	126 (64.9%)
Low to moderate	50 (25.8%)
Moderate to severe	11 (5.7%)
Severe to extreme	7 (3.6%)
Physical Neglect	6.61 (±2.32)
None to minimal	145 (74.7%)
Low to moderate	32 (16.5%)
Moderate to severe	12 (6.2%)
Severe to extreme	5 (2.6%)
Inconsistency	5.12 (±2.78)
Playing down	0.820 (±1.00)

Shown as either n (% of N) or Mean (± standard deviation).

### Mediation model

To test for significant mediation of the effect of CTQ on EAT *via* the serial mediators BDI and physical exercise (see Figure 1 for our model), we tested presence of a completely standardized indirect effect using bias-corrected bootstrapping with 10,000 resamples and a 95% CI.

**Figure 2:**
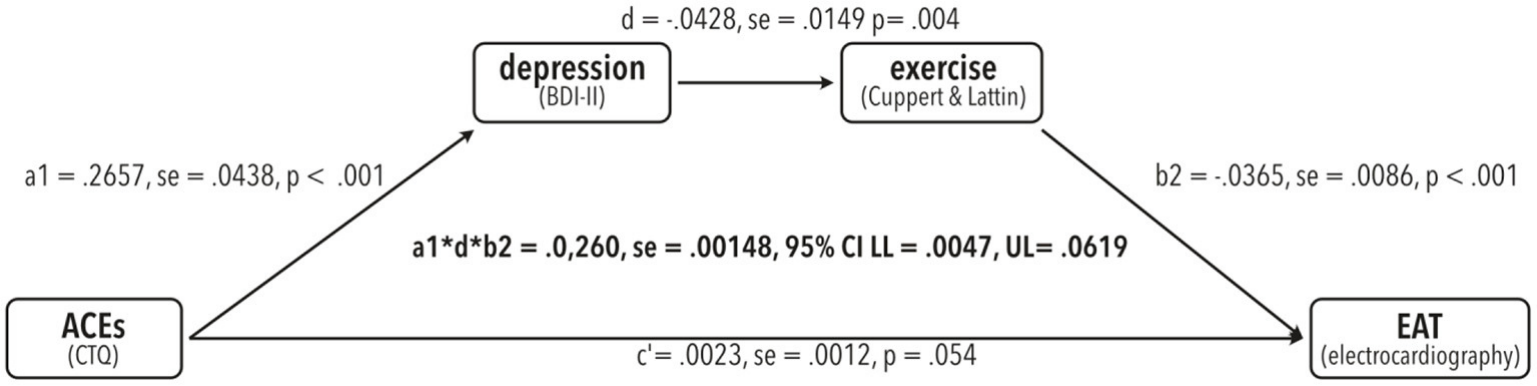
Results from the testing the serial mediation model (model number 6) proposed in Figure 1 using bias-corrected bootstrapping with 10,000 resamples in PROCESS for R V4.0.1 with a 95% confidence interval (CI). Unstandardized regression coefficients are shown with the corresponding se (standard error) as well as t-values and *p*-values per path. Indirect effect statistics are shown in the center with completely standardized regression coefficients, se and corresponding lower limits (LL) and upper limits (U).

There was a significant indirect effect of CTQ on EAT via depression and exercise (a1^*^d^*^b2 = 0.0260, se = 0.00148, 95%CI: LL = 0.0047, UL = 0.0619), meaning the effect of CTQ on EAT was mediated *via* the links CTQ -> BDI, BDI -> exercise and exercise -> EAT (Also compare Figure 2).

### Additional regression analyses

**Figure 3:**
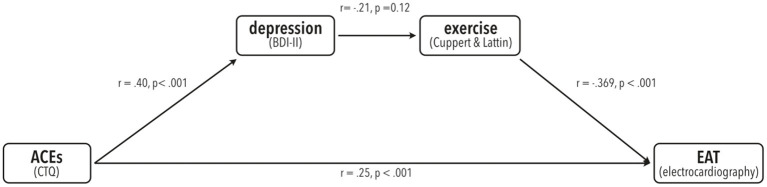
Illustration of the results from single linear regression analyses from all paths present in the mediation model. Pearson's r-values and corresponding *p*-values are shown.

Although usage of the mediation procedure developed by Baron and Kenny (57) that employs single regression analyses has been proven invalid and is no longer advised (58, 59), we conducted additional regression analyses to illustrate the relationships between the variables used in our mediation model. All these relationships were significant as described in the following and the same mediation effect was found when using Baron and Kenny criteria (see Figure 3). Please note that presence or absence of such links is neither necessary nor meaningful in the analysis of mediation, which can be measured using single bootstrapping techniques (53, 59). Nonetheless, we chose to show these data for an easier understanding of the relationships.

## Discussion and limitations

In this study, we examined the relationship between adverse childhood experiences (ACE) and their link to increased cardiovascular risk in adulthood. The results of the statistical analysis support the hypothesis that ACE path the way to the development of depressive symptoms. Loss of energy and anhedonia are important symptoms of depression, therefore one could argue that the reduction of physical activity may be the consequence of depressive psychopathology. The net effect of depression plus physical inactivity may then foster the development of epicardial adipose tissue over time. This temporal sequence is in line with the allostasis model (60, 61), which describes the cumulative effects of experiences in life that involve ordinary events as well as major challenges, resulting in physiological changes and health jeopardizing behaviors (35).

ACE, *via* the pathway of depression and subsequent reduced physical exercise, correlates with an increase in epicardial adipose tissue and, therefore, risk for adverse cardiac outcomes.

This pathway opens room for interventions on all levels of prevention and may be considered within the framework of preventative medicine. Future research may focus on primary prevention of ACE altogether—an intervention which will likely need to happen on a societal level and appears even more important in view of the consequences of ACE for cardiovascular health.

Secondary prevention may focus on avoiding the development of depressive symptoms in those who have suffered from ACE. In this view, secondary prevention may not only improve the quality of life of those who suffered ACE, but also reduce the risk of psychiatric and cardiovascular morbidity in later life.

Once depression has developed, the pathway described above implies that special interest of tertiary prevention may be the increase of physical activity. These patients may profit doubly from approaches focusing on the reduction of loss of energy–once in terms of depression and once in terms of a reduction in cardiovascular risk. Thus, preferred medications may be those which are effective and have a favorable side effect profile, i.e., low risk of gaining weight. Psychotherapeutic interventions that increase activity, such as cognitive behavioral therapy (62), acceptance and commitment treatment (54, 63), or behavioral activation (64, 65), have shown efficacy in the treatment of depression but may also be beneficial for cardiac health although evidence in this regard is currently lacking. Other psychological interventions have also shown effectiveness particularly in patients with underlying ACE, such as the cognitive behavioral analysis system of psychotherapy (CBASP) (66, 67).

Of course, this pathway shows the behavioral level of connection between ACE and EAT and questions as to the biological mechanisms behind it remain mostly unclear. Part of the increased cardiovascular risk after ACE may be explained through HPAA-activation. Former studies from our center linked ACE to hypercortisolism with a consecutive increase of adrenal gland volume as mediating factors for EAT (68, 69). Further research may however incorporate e.g., epigenetic factors and their link to observable behavior as well to generate a more complete understanding of the mechanisms behind this behavioral pathway.

The results of our study add to the literature in that ACEs have long-term effects on mental and physical health, and on health behavior. Hughes et al. found in their systematic review that multiple ACE pose a major risk for many health conditions, and, especially, for next-generation ACE (35).

Other studies described marked changes in the HPAA both in terms of lowered and elevated cortisol levels. A meta-analysis by Klaassens et al. (38) found that while neither adulthood exposure to trauma nor PTSD changed HPAA-functioning, they significantly augmented cortisol suppression. Khoury et al., in yet another meta-analysis evaluating the association between ACE and hair cortisol levels (39), suggested that there is in fact a hyperactivity of the HPAA that due to neurodepletion forms into a hypoactivity and thereby lowered cortisol levels. These changes in cortisol secretion have hitherto been linked to a marked increase in cardiovascular risk (35, 70). Pilkington et al. found in their meta-analysis that ACE, especially emotional neglect, correlated with maladaptive schemas (24)—which in turn contribute to various psychological problems, including depression (11, 71).

This study's results point toward a definite behavioral pathway that links ACE to CVD-risk by means of depression and consecutive physical inactivity and, thus, provides insight into possible points of intervention.

Previous studies using mediation analysis to examine the pathway linking ACE to cardiovascular risk, support this study's findings. A report of the Whitehall II study cohort by Deschênes et al. found that ACE were associated with a higher risk of diabetes *via* the pathway of depressive symptoms or cardiometabolic dysregulation (34). Slopen et al., complementary to this study, found that positive childhood experiences were connected to cardiovascular health, mediated by depressive status and social support (40). Kraynak et al. found a possible pathway between childhood physical abuse and corticolimbic activity (specifically that between amygdala, ACC and vmPFC) mediated by systemic inflammatory response (IL-6 levels). As there is a connection between long-term IL-6 levels and physical activity (72), the pathway shown in this study may extend the Kraynak's findings. Further research in mediating factors may complete the pathway that links ACE to depressive symptoms, neuroendocrinological dysfunction, systemic inflammatory response and cardiovascular health.

Limitations of this study exist. Firstly, there is the general limitation of a relatively small sample size and the herewith connected question of representation of the studied sample for a more general population. While we aimed to correct this by means of bootstrapping, further studies replicating the findings of this study are needed to confirm the pathway.

Secondly, there is the issue as to the selection of patients studied. The sample included only patients with congenital heart disease and may therefore not be representative for a broader population. Not in the least, because of the major impact that the diagnosis of a congenital heart disease has on the family system, potentially causing an atmosphere of anxiety and insecure attachment and, thus, harboring ACEs. Interestingly, both amount of ACE and their severity as reported by this study's subjects were relatively low (which, duly noted, implies that there, at least, was no over-reporting due to the retrospective collection of data on ACE). No more than 6% of the patients reported ACE corresponding with moderate to severe scores and even on the scale of emotional neglect, only 35.1% answered above the cut-off value. Hence, there is a slim possibility that the mediated effect may be a coincidental result or that a greater effect of ACE remains masked.

Thirdly, the retrospective report of childhood trauma may have lead to recall and other kinds of memory or reporting biases (73, 74). Hence, a prospective longitudinal study that examines the relationship between ACE and later health, especially with a focus on the development of psychiatric morbidity and connected cardiovascular morbidity, may provide further insight into the mechanisms that connect ACE with cardiovascular risk. We do, however, believe that the patient's individual perception of their childhood experiences as traumatic, regardless of whether or not external observers would rate them as such, plays a major role for the effects that ACE have on the patient's later biography. We thus believe it justified to use a retrospective self-report questionnaire.

In summary, we found an association of ACE to cardiovascular risk (in terms of increased EAT) which was mediated by means of depression and subsequent reduction of physical activity, and have thereby shown a first behavioral pathway that links ACE to cardiovascular health. While there are some limitations mainly due to statistical power (which was corrected by bootstrapping), and potential selection bias (too low CTQ-values, only ACHD patients), these findings are robust–especially in view of the logical soundness of the hypothesis.

## Data availability statement

All data supporting the conclusions of this article will be made available by the authors upon request without undue reservation.

## Ethics statement

The studies involving human participants were reviewed and approved by Ethics Commission of Hanover Medical School. The patients/participants provided their written informed consent to participate in this study.

## Author contributions

The text was primarily written (in equal shares) by IH and SB. All authors listed contributed substantially to this study's conception, design, and performance. All authors contributed to the article and approved the submitted version.

## Conflict of interest

The authors declare that the research was conducted in the absence of any commercial or financial relationships that could be construed as a potential conflict of interest.

## Publisher's note

All claims expressed in this article are solely those of the authors and do not necessarily represent those of their affiliated organizations, or those of the publisher, the editors and the reviewers. Any product that may be evaluated in this article, or claim that may be made by its manufacturer, is not guaranteed or endorsed by the publisher.
